# The Use of Upcycled Defatted Sunflower Seed Flour as a Functional Ingredient in Biscuits

**DOI:** 10.3390/foods8080305

**Published:** 2019-08-01

**Authors:** Simona Grasso, Ese Omoarukhe, Xiaokang Wen, Konstantinos Papoutsis, Lisa Methven

**Affiliations:** 1 School of Agriculture, Policy and Development, University of Reading, Reading RG6 6AR, UK; 2 School of Agriculture and Food Science, University College Dublin, D04 V1W8 Dublin, Ireland; 3 Department of Food and Nutritional Sciences, University of Reading, Reading RG6 6AP, UK

**Keywords:** biscuits, upcycled food by-products, defatted sunflower seed flour, sensory QDA, TPA, colour, antioxidant capacity, protein enrichment, functional foods, valorisation

## Abstract

Defatted sunflower seed flour (DSSF) is an upcycled by-product of sunflower oil extraction, rich in protein, fibre and antioxidants. This study assessed the instrumental and sensory quality of biscuits enriched with DSSF at 18% and 36% *w*/*w* as a replacement for wheat flour. Measurements included colour, texture, total phenolic content (TPC) and antioxidant capacity. Sensory analysis was carried out with Quantitative Descriptive Analysis (QDA). The inclusion of DSSF significantly increased the protein content of the biscuits, as well as the TPC and antioxidant capacity of the biscuits. The resulting products were significantly darker, less red and less yellow with increasing DSSF levels, while hardness (measured instrumentally) increased. Sensory results agreed with colour measurements, concluding that DSSF biscuits were more “Brown” than the control, and with texture measurements where biscuits with 36% DSSF had a significantly firmer bite. In addition, DSSF biscuits at 36% inclusion had higher QDA scores for “Off-note” and the lowest scores for “Crumbly” and “Crumb aeration”. DSSF biscuits at 18% inclusion were similar to the control in most parameters and should be considered for further developments. These results show the potential of the upcycled DSSF by-product as a novel, sustainable and healthy food ingredient.

## 1. Introduction

Sunflower (*Helianthus annuus* L.) is one of the three most cultivated oil crops in the world [1]. The main by-product of the oil extraction process, which can constitute up to 36% of the mass of the processed seeds [1], is the so-called sunflower meal or cake. This by-product has a high protein content (40–50%) [2] and is used primarily in ruminant feed [1].

The sunflower cake contains essential amino acids (such as lysine, methionine, cystine, tryptophan), minerals, B group vitamins [1] and has a high antioxidant potential [3], making this product interesting as human food. On the other hand, some limitations include a high insoluble fibre content, the residue solvents used for oil extraction in the cake [3] and the presence of anti-nutrients such as protease inhibitors, saponins and arginase inhibitor [4]. 

Steam explosion, involving high pressure and high temperature, has recently been used on various substrates and by-products to break insoluble fibre into smaller soluble dietary fibre units [5,6], to decompose some anti-nutrients [7] and as a sterilisation method [8]. The US company Planetarians uses steam explosion on sunflower cake to produce a commercially available food grade defatted sunflower seed flour (DSSF) without the need for purification steps [9]. Within the context of circular bio-economy, there is a growing interest in the food industry to use inexpensive upcycled by-products to partially replace flour, fat or sugar in bakery products to achieve value-added, nutritionally-enriched and sustainable foods [10]. 

The aim of the present work is to use DSSF in biscuits, substituting it for 18% and 36% of wheat flour, and investigating the effects that the DSSF inclusion might have on the quality of the biscuits, both from an instrumental and sensory point of view. 

## 2. Materials and Methods 

### 2.1. Materials

Wheat flour (composition from manufacturer: fat 1.7%, carbohydrate 74%, fibre 3.8%, protein 9.9%), sugar, sunflower oil, cocoa powder, sodium bicarbonate and sodium chloride used for biscuit formulations were supermarket own-label from a local retailer. The DSSF (composition from manufacturer: fat 1%, carbohydrate 48%, fibre 18%, protein 35%), obtained after steam explosion and milling, was donated by the company Planetarians (Palo Alto, CA, USA).

### 2.2. Biscuit Preparation

Short dough biscuits were prepared according to the modified method of Kuchtová, Karovičová, Kohajdová, Minarovičová and Kimličková [10]. Control biscuits were manufactured without DSSF, while DSSF biscuits were made substituting respectively 18% and 36% of wheat flour for DSSF. In the control recipe, the oil (26.5 g) and sugar (35 g) were mixed at medium speed for 5 min using a mixer (Major Titanium KM020, Kenwood, London, UK). Water (62 g) was added and mixed for 30 s at low speed. Then the remaining ingredients (flour 90 g, cocoa 10 g, sodium bicarbonate 1.1 g and salt 0.9 g) were added and mixed for 2 min at low speed. In the 18% and 36% DSSF recipes, respectively 18% of flour (16.4 g) or 36% of flour (32.8 g) was replaced with DSSF. The dough was sheeted to a 4 mm thickness with a Rondo table model dough sheeter (Rondo, Burgdorf, Switzerland) and cut by hand with a 55 mm diameter round cutter. The biscuits were baked on aluminium trays in a ventilated oven (Kwick_Co, Salva, Gipuzkoa, Spain) for 15 min at 190 °C. After 30 min of cooling time, the biscuits were vacuum packed and stored until further analysis. 

### 2.3. Proximate Analysis

The moisture, protein, fat and ash content of the wheat flour, DSSF and biscuits were determined using methods from the Association of Official Analytical Chemists (AOAC) [11]. The total content of carbohydrates was calculated by difference: 100 − (moisture + ash + protein + fat). 

### 2.4. Determination of Total Phenolic Content and Antioxidant Capacity

The extracts for the determination of total phenolic content (TPC) and antioxidant capacity were prepared according to Ajila et al. [12] with some modifications. Briefly, 1 g of sample was mixed with 20 mL of absolute methanol and left at ambient temperature for 1 h. Subsequently, the mixture was centrifuged (1.5× *g*, 10 min) and the supernatant was collected and used for the determination of TPC and antioxidant capacity.

#### 2.4.1. Total Phenolic Content (TPC)

TPC was measured for the DSSF, wheat flour and biscuits according to Singleton and Rossi [13] with some modifications. Briefly, 2.5 mL of 10% (*v*/*v*) Folin–Ciocalteu reagent was mixed with 0.5 mL of sample. After 3 min of incubation, 2 mL of 7.5% (*w*/*v*) Na_2_CO_3_ was added to the mixture and incubated in the dark at room temperature for 1 h. The absorbance of the solution was measured at 765 nm. The results were expressed as mg of gallic acid equivalents per g of sample dry weight (mg GAE/g).

#### 2.4.2. Antioxidant Capacity

The 2,2-Diphenyl-1-picrylhydrazyl (DPPH) radical scavenging capacity was determined according to Vamanu and Nita [14] and Papoutsis et al. [15]. Briefly, 2850 mL of DPPH solution was mixed with 150 μL of extract. The mixture was left to stand for 30 min in the dark. The absorbance was measured at 515 nm. Results were expressed mg Trolox equivalents per g (mg TE/g).

The cupric reducing antioxidant capacity (CUPRAC) was determined according to Apak et al. [16] with some modifications. Briefly, 1 mL of 10 mM copper chloride (II) was mixed with 1 mL of 7.5 mM neocuproine solution and 1 mL of NH_4_Ac buffer (pH 7.0). Subsequently, 1.1 mL of sample was added to this mixture. The mixture was incubated at room temperature for 1.5 h before measuring the absorbance at 450 nm. Results were expressed as mg Trolox equivalents per g (mg TE/g).

### 2.5. Physical Analyses

The width and thickness of at least 10 biscuits per batch and per recipe were measured with a digital calliper after baking. The spread ratio of the biscuits was calculated dividing width by thickness [17].

Hardness was measured using a texture analyser TA-XT2i (Stable Micro Systems, London, UK) equipped with a three-point bending rig (HDP/3PB). Hardness was the maximum resistance of each biscuit against a rounded edge blade and occurred when the sample began to break.

Water holding capacity (WHC) and oil-adsorption capacity (OAC) was measured for wheat flour and DSSF. WHC was determined according to Sudha et al. [18] with slight modifications. Aliquots of 0.05 g of DSSF or wheat flour were mixed with 1 mL water in a microcentrifuge tube, centrifuged at 13,000× *g* for 30 min, and the excess water was decanted. The sample was weighed, and WHC was expressed as g water/g dry weight. OAC was similarly determined, by using sunflower oil instead of water. OAC was expressed as g oil/g dry weight.

### 2.6. Colour

The colour of the wheat flour, DSSF and biscuits was measured using a colorimeter (CR-400, Konica, Minolta, Japan), calibrated using a white standard plate. The values measured were L* (white 100/black 0), a* values (red positive/green negative) and b* values (yellow positive/blue negative). Colour was measured for 10 biscuits in each batch. The total colour difference (ΔE) was calculated according to the equation:

ΔE = [(a* − a_0_*)^2^ + (b* − b_0_*)^2^ + (L* − L_0_*)^2^]^1/2^

### 2.7. Sensory Evaluation

Sensory profiling of biscuits was conducted by a panel of nine trained panellists (eight female, one male, mean age 47 years). A consensus vocabulary of 29 descriptors was developed to characterise the samples; under the modalities appearance (5), aroma (4), taste and flavour (8), mouthfeel (5) and after-effect descriptors (7), using reference standards where required. The purpose of the sensory profiling was to provide a consistent measure for changes in biscuit descriptors occurring with change in formulation. Descriptor scoring was done using unstructured line-scales (scale 0–100) using the Compusense^®^ software (Compusense, ON, Canada). Panellists were seated in individual testing booths under artificial daylight. Samples (one biscuit per person per sample) were presented in a balanced order, randomly allocated and single-blinded using three-digit number codes. Panellists were asked to taste at least half of the portion size. Warm filtered water was used as a palate cleanser and the time delay between samples (post after-effects scoring) was 30 s. Biscuit scoring was carried out in duplicates on two consecutive days.

### 2.8. Statistical Analysis

For instrumental measurements, the experiment was repeated three times on three different days. Statistical analyses were carried out using analysis of variance (ANOVA) or independent t-tests with the software SPSS (V24, SPSS Inc., Chicago, IL, USA). The determination of significance among the control, 18% DSSF, and 36% DSSF biscuits was conducted by Tukey’s post hoc multiple comparison test at a significance level of *p* < 0.05. For sensory data, a two-way ANOVA was used. The panellists were fitted as random effects and the samples were fixed effects. The treatment effects (samples and assessors) were tested against the panellist by assessor interaction.

## 3. Results and Discussion

### 3.1. Physical and Chemical Properties of Defatted Sunflower Seed Flour

The physical and chemical properties of wheat flour and DSSF are reported in Table 1. The DSSF ingredient presented a lower moisture and a fourfold higher protein content compared to wheat flour. Fat and ash content were also significantly higher compared to wheat flour.

In terms of colour, DSSF was significantly darker than wheat flour, with higher redness and blueness values. The hydration properties of DSSF fell in the same range reported by other authors on apple pomace [18,19]. DSSF presented a threefold higher WHC than wheat flour, possibly due to the high content of soluble dietary fibre. Ash content is a good indicator of mineral content and according to the literature [20], sunflower oil cake on a dry basis contains 0.48% calcium, 0.84% phosphorus, 0.44% magnesium and 3.49% potassium. 

**Figure 1:**
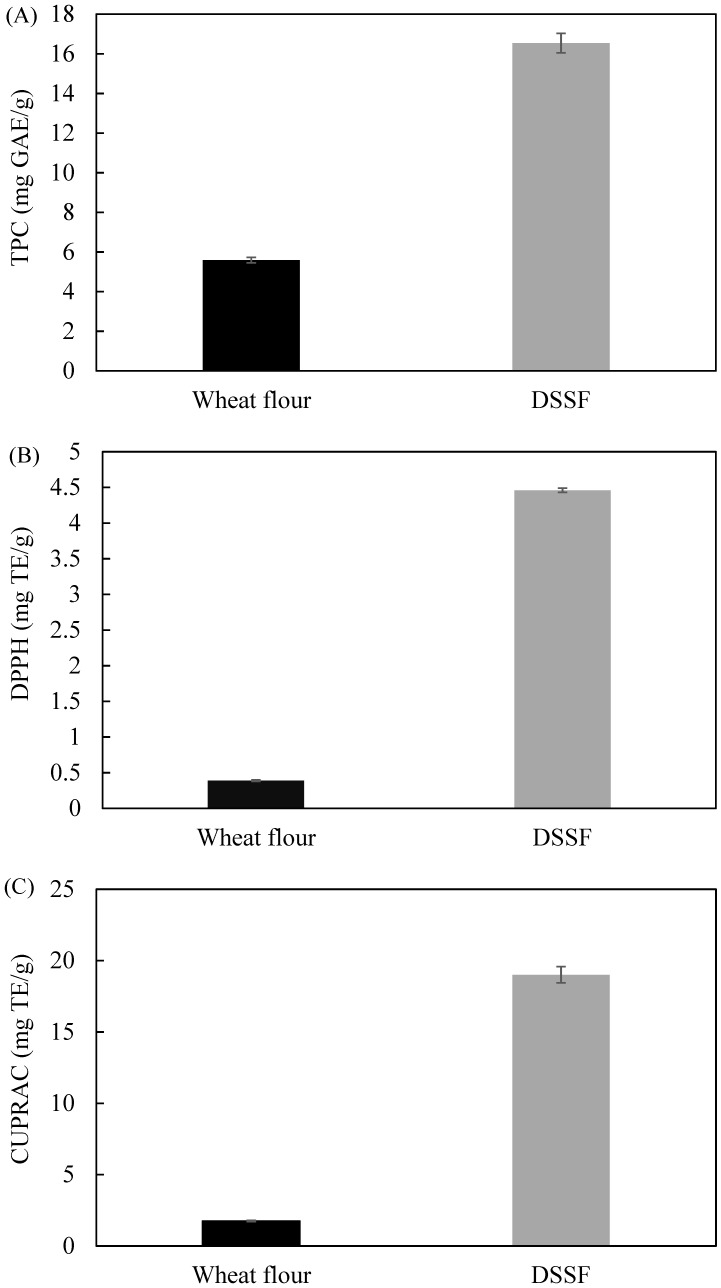
Total phenolic content (TPC) (**A**) 2,2-Diphenyl-1-picrylhydrazyl (DPPH) (**B**) and cupric reducing antioxidant capacity (CUPRAC) (**C**) values of wheat flour and defatted sunflower seed flour (DSSF). Data are expressed as means ± SD (*n* = 3).

Results for TPC, DPPH and CUPRAC are shown in Figure 1. TPC in DSSF was 16.54 mg GAE/g, while TPC for wheat flour was significantly lower at 5.59 mg GAE/g. These values are higher than those reported on apple pomace (1.1 mg GAE/g dry weight) [19] and beetroot pomace (up to 3.8 mg GAE/g dry weight) [21]. Similarly to TPC results, DSSF had higher antioxidant capacity measured by DPPH and CUPRAC assays compared to wheat flour. Previous studies have shown that sunflower flour is a good source of phenolic compounds including chlorogenic, caffeic, p-hydroxybenzoic, *p*-coumaric, cinamic, m-hydroxybenzoic, vanillic, syringic, transcinnamic, isoferulic and sinapic acids, which are compounds with high antioxidant capacity [22]. On the other hand, wheat flour has been reported to have very low polyphenol content [23], which justifies its lower antioxidant capacity compared to DSSF.

### 3.2. Effect of Defatted Sunflower Seed Flour on the Physical and Chemical Properties of Biscuits

The physical properties of biscuits made replacing wheat flour with 18% and 36% of DSSF are presented in Table 2. When compared to control biscuits, the diameter of DSSF biscuits at both inclusion levels was significantly lower. Thickness also decreased at the 36% DSSF inclusion level, while there was no significant difference in thickness between the control and the 18% DSSF biscuits. The decrease in thickness and diameter in biscuits with DSSF inclusion might be due to the dilution of gluten [17] or increase in fibre content [24] and is in agreement with similar studies on by-product incorporation [10,19]. Cookie diameter is considered a quality indicator and cookies with larger diameters are usually more desirable [25]. 

There was no significant difference in spread ratio between the control and the 18% DSSF biscuits, while the 36% DSSF biscuits had a significantly higher spread ratio. This might be due to the higher fat content. As explained by Kuchtová, Karovičová, Kohajdová, Minarovičová and Kimličková [10], an increase in fat content leads to an increase in spread ratio, which might be due to the higher fat content in the by-product. Usually the higher the spread ratio of the biscuit, the more desirable it is [26].

DSSF biscuits were harder than the control, which is in contrast to similar studies on by-product incorporation such as apple pomace or grape pomace in biscuits [10,19]. This might be due to the fact that DSSF has a very high protein content compared to other by-products such as apple pomace or grape pomace, which might contribute to hardness. The contribution of protein content to biscuit hardness has been previously reported with whey protein concentrates and defatted soy flour addition [27,28].

Lightness decreased significantly with increasing DSSF inclusion levels. This was expected as DSSF is darker in colour compared to wheat flour, as seen in Table 1. The same pattern can be seen for a* and b*, as these parameters also significantly decreased with increasing DSSF addition, indicating a more intense green and less intense yellow colour in DSSF biscuits compared to the control. As expected, the ΔE, representing the overall difference in colour compared to the control, increased with increasing DSSF addition. These results agree with those from Kuchtová, Karovičová, Kohajdová, Minarovičová and Kimličková [10], Bhat and Hafiza [25] and de Toledo et al. [29], reporting colour alterations in biscuits enriched with by-products. 

### 3.3. Effect of Defatted Sunflower Seed Flour on the Proximate Conmposition and Chemical Properties of Biscuits

The proximate composition of control and DSSF biscuits is shown in Table 3. Protein, fat and ash content were significantly higher in DSSF biscuits compared to the control, while the carbohydrate content was lower. There was also a significant difference between DSSF biscuits, with the 36% DSSF biscuits showing the highest protein and ash values between the two, due to the higher DSSF percentage of inclusion. Biscuits with 36% DSSF could be labelled as a “source of protein”, because at least 12% of the biscuit calories come from protein [30].

**Figure 2:**
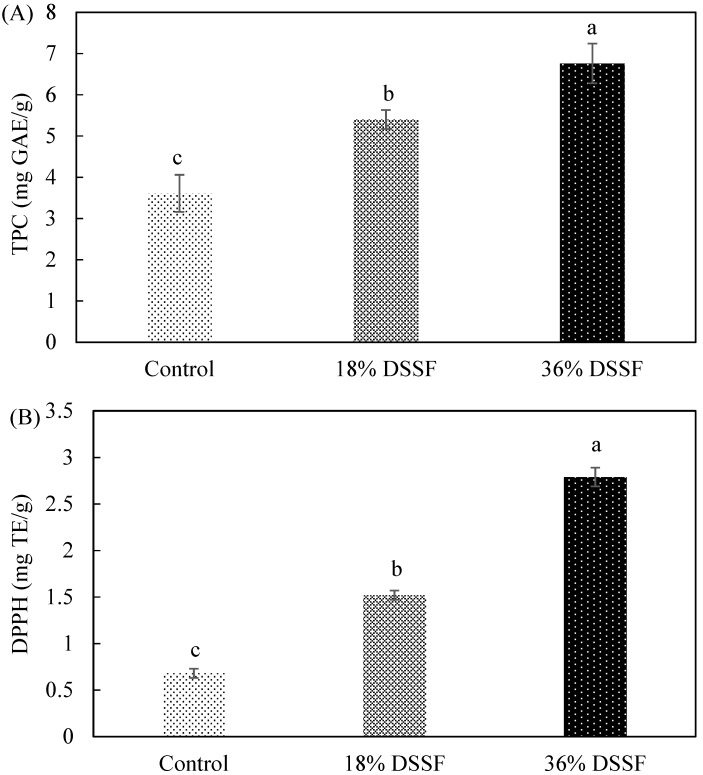

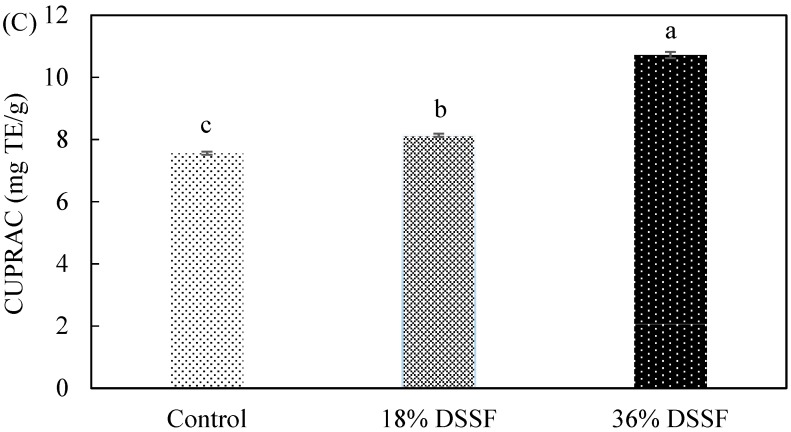
Total phenolic content (TPC) (**A**), DPPH (**B**), and CUPRAC (**C**) values of control and defatted sunflower seed flour (DSSF) biscuits at 18% and 36% inclusion. Data are expressed as means ± SD (*n* = 3). Bars with same letter are not significantly different at *p* < 0.05.

TPC results (Figure 2A) show that control biscuits had the lowest phenolic content, while DSSF biscuits had significantly higher TPC values. The DPPH and CUPRAC assays (Figure 2B,C) show significant differences among the three recipes, with antioxidant capacity being lowest in the control and then increasing significantly with increasing DSSF inclusion. The higher antioxidant capacity of the 36% DSSF biscuits can be explained by the higher TPC content, since high correlation between antioxidant capacity and phenolic compounds has been reported [31]. Similar results have been reported by Gbenga-Fabusiwa et al. [32], who found that biscuits produced from pigeon pea–wheat flour had higher phenolic content and antioxidant activities compared to those produced with wheat flour only. Aksoylu et al. [33] reported higher TPC in biscuits made with blueberry and grape seeds, while Ajila, Leelavathi and Rao [12] reported that biscuits with mango peel powder had higher DPPH activity. 

### 3.4. Effect of Defatted Sunflower Seed Flour on Sensory Properties of Biscuits

**Figure 3:**
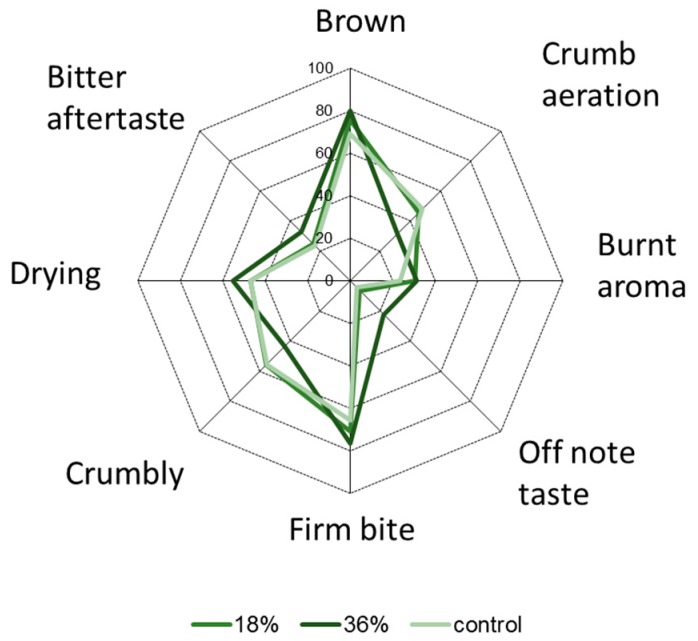
Sensory scores of descriptors that differed significantly between biscuits (control, 18% and 36% defatted sunflower seed flour).

The trained panel detected significant differences in eight descriptors of the 29 rated (Figure 3 and Table 4). In terms of appearance, panellists scored DSSF biscuits as more brown than the control, which is in accordance with the instrumental colour test results. Another appearance descriptor that differed was crumb aeration, with the 18% DSSF and control biscuits showing similar crumb aeration, while the 36% DSSF biscuits showed a less aerated crumb. This also corresponds to instrumental measurements, which showed that the 36% DSSF biscuits were significantly less thick compared to the control and 18% DSSF biscuits. 

The only aroma descriptor that differed between the samples was burnt aroma. This descriptor scored significantly higher in DSSF biscuits compared to the control, probably due to an acceleration of the Maillard reaction rate in DSSF biscuits [34]. This could be related to the higher amino acid content and the lower sugars in DSSF. Similar results for the descriptor baked flavour were reported by Alongi, Melchior and Anese [19] where apple pomace at 18% and 36% inclusion was added in biscuits. 

The only taste descriptor that was significantly different among the three biscuit recipes was off-note. Significantly higher off-note scores were found in the 36% DSSF biscuits, while the control and 18% DSSF biscuits were similar in this parameter. These results could be associated with the bitter and astringent taste of by-products, which is due to the high phenolic content [10,35]. On the other hand, the addition of DSSF even at 36% did not significantly affect the sweet taste descriptor scores, which is a positive finding when compared to the decrease in sweetness reported by Davidov-Pardo et al. [36] in cookies with grape seed extracts.

In terms of texture, 36% DSSF biscuits were significantly harder than 18% DSSF and control biscuits, which concurs with results from instrumental measurements. For the descriptor crumbly, control and 18% DSSF biscuits scored similarly, while 36% DSSF biscuits scored significantly lower, possibly indicating that these biscuits were more compact, less aerated and therefore behaved differently during mastication. Interestingly, the descriptor grainy in relation to biscuit texture was never used by the panellists, while similar studies on incorporation of grape, blueberry and poppy by-products reported grainy textures and rough structures in biscuits [10,33]. The small particle size of DSSF (US mesh 100) might have been beneficial in preventing issues related to graininess.

Finally, differences were perceived on two after effects descriptors, drying and bitter. Again 36% DSSF biscuits scored higher values in these descriptors compared to control and 18% DSSF biscuits. The abundant phenolic content of DSSF could be responsible for observed drying and bitter aftertaste in 36% DSSF biscuits. Upon consumption, the phenolic compounds in DSSF may interact with the glycoproteins in saliva, resulting in less saliva being available to dissolve the biscuits and spread the fat in the mouth [36]. 

In general, the inclusion of DSSF in biscuits led to sensory changes, more noticeable at high inclusion levels of 36%, but not as much with the lower 18% inclusion. Similar conclusions were reached by Alongi, Melchior and Anese [19]. These authors observed significant changes to the sensory profile of biscuits where wheat flour was replaced with 20% apple pomace, while with a lower concentration of 10% no changes were perceived compared to the control. Similarly, de Toledo, Nunes, da Silva, Spoto and Canniatti-Brazaca [29] found that replacing up to 14% of wheat flour with pineapple, apple and melon by-products did not result in significant sensory differences, while higher inclusion levels did. 

## 4. Conclusions

This study concluded that where upcycled DSSF was used to replace flour in a short-dough biscuit, the protein content substantially increased, as did the antioxidant capacity and TPC of the biscuits. An 18% replacement of wheat flour with DSSF led to products that were significantly different from the control in only two attributes (brown colour and burnt aroma). The 36% inclusion resulted in biscuits that were significantly less crumbly, less aerated, with a higher off-note, higher drying and bitter after taste compared to both the 18% DSSF and control biscuits. Future work could focus on reformulation aiming to test smaller inclusion levels at 9% and 27% and to test the use of additional ingredients to optimize the recipe. 

## Figures and Tables

**Table 1:** Physical and chemical properties of defatted sunflower seed flour and wheat flour.

Parameters		Wheat Flour	DSSF
Moisture (%)		10.22 ± 0.02 ^a^	4.59 ± 0.02 ^b^
Protein (%)		9.8 ± 0.02 ^b^	38.01 ± 0.01 ^a^
Fat (%)		1.62 ± 0.01 ^b^	1.84 ± 0.03 ^a^
Ash (%)		0.94 ± 0.05 ^b^	7.19 ± 0.03 ^a^
WHC (g water/g dry weight)		0.69 ± 0.14 ^b^	2.21 ± 0.18 ^a^
OAC (g oil/g dry weight)		0.87 ± 0.03 ^b^	1.25 ± 0.06 ^a^
Colour	L*	93.93 ± 0.36 ^a^	62.99 ± 0.12 ^b^
	a*	−0.79 ± 0.06 ^b^	1.47 ± 0.02 ^a^
	b*	11.45 ± 0.15 ^b^	14.38 ± 0.07 ^a^

Data are expressed as means ±SD of duplicate or triplicate assays. Values with the same letter in the same row are not significantly different at *p* < 0.05. DSSF, defatted sunflower seed flour; OAC, oil-adsorption capacity; WHC, water holding capacity.

**Table 2:** Physical properties of control and defatted sunflower seed flour biscuits.

Parameters		Control	18% DSSF	36% DSSF
Diameter (mm)		55.15 ± 1.26 ^a^	54.18 ± 1.03 ^b^	53.9 ± 0.91 ^b^
Thickness (mm)		8.66 ± 0.40 ^a^	8.62 ± 0.27 ^a^	8.13 ± 0.25 ^b^
Spread ratio		6.38 ± 0.35 ^b^	6.29 ± 0.24 ^b^	6.64 ± 0.26 ^a^
	L*	38.10 ± 0.88 ^a^	37.40 ± 0.58 ^b^	36.55 ± 0.42 ^c^
Colour	a*	6.8 ± 0.26 ^a^	4.96 ± 0.26 ^b^	3.93 ± 0.28 ^c^
	b*	5.16 ± 0.60 ^a^	4.49 ± 0.39 ^b^	3.87 ± 0.37 ^c^
Delta E		-	4.3	11.9
Hardness (N)		40.98 ± 5.44 ^b^	50.25 ± 5.53 ^a^	53.27 ± 7.10 ^a^

Data are expressed as means ±SD on at least 10 biscuits for each of the three batches. Values with the same letter in the same row are not significantly different at *p* < 0.05.

**Table 3:** Proximate composition of control and defatted sunflower seed flour biscuits.

Parameters	Control	18% DSSF	36% DSSF
Carbohydrate (%)	69.56 ^a^	65.16 ^b^	61.42 ^c^
Fat (%)	17.37 ± 0.5 ^b^	18.33 ± 0.1 ^a^	18.47 ± 0.4 ^a^
Protein (%)	7.98 ± 0.08 ^c^	10.80 ± 0.12 ^b^	13.61 ± 0.18 ^a^
Ash (%)	2.18 ± 0.05 ^c^	2.68 ± 0.03 ^b^	3.27 ± 0.04 ^a^
Estimated calories (Kcal/100 g)	465	467	464
Calories from protein (%)	7	9	12

Data are expressed as means ± SD of duplicate assays. Values with the same letter in the same row are not significantly different at *p* < 0.05.

**Table 4:** Sensory scores of control and defatted sunflower seed flour biscuits.

Parameters	Descriptor	Control	18% DSSF	36% DSSF	*p*-Value
Appearance	Brown	69.4 ^b^	75.6 ^a^	80.3 ^a^	0.0024
	Crumb aeration	48.2 ^a^	45.4 ^a^	31.3 ^b^	0.0001
Aroma	Burnt	23.6 ^b^	30.6 ^a^	31.3 ^a^	0.0390
Taste and flavour	Off note	4.1 ^b^	6.3 ^b^	22.3 ^a^	0.0162
Mouthfeel	Firm bite	65.5 ^b^	70.7 ^ab^	76.2 ^a^	0.0313
	Crumbly	55.5 ^a^	56.0 ^a^	43.6 ^b^	0.0219
After effects	Drying	46.7 ^b^	46.9 ^b^	55.0 ^a^	0.0053
	Bitter	23.5 ^b^	24.8 ^b^	32.5 ^a^	0.0385

Data are expressed as means of duplicate scoring sessions. Values with the same letter in the same row are not significantly different at *p* < 0.05.

## References

[1] Yegorov B., Turpurova T., Sharabaeva E., Bondar Y. (2019). Prospects of using by-products of sunflower oil production in compound feed industry. J. Food Sci. Technol. Ukr..

[2] González-Pérez S., Merck K.B., Vereijken J.M., van Koningsveld G.A., Gruppen H., Voragen A.G.J. (2002). Isolation and Characterization of Undenatured Chlorogenic Acid Free Sunflower (Helianthus annuus) Proteins. J. Agric. Food Chem..

[3] Wanjari N., Waghmare J. (2015). Phenolic and antioxidant potential of sunflower meal. Adv. Appl. Sci. Res..

[4] Francis G., Makkar H.P.S., Becker K. (2001). Antinutritional factors present in plant-derived alternate fish feed ingredients and their effects in fish. Aquaculture.

[5] Zhang X., Han G., Jiang W., Zhang Y., Li X., Li M. (2016). Effect of Steam pressure on chemical and structural properties of kenaf fibers during steam explosion process. BioResources.

[6] Jung J.Y., Heo J.M., Yang J.K. (2019). Effects of steam-exploded wood as an insoluble dietary fiber source on the performance characteristics of Broilers. BioResources.

[7] Gu X., Dong W., He Y. (2011). Detoxification of Rapeseed Meals by Steam Explosion. J. Am. Oil Chem. Soc..

[8] Zhao Z.M., Wang L., Chen H.Z. (2015). A novel steam explosion sterilization improving solid-state fermentation performance. Bioresour. Technol..

[9] Planetarians White Paper. Planetarians: Helping Companies to Find Better Ingredients for People and the Planet. https://www.planetarians.com/planetarians-technology.

[10] Kuchtová V., Karovičová J., Kohajdová Z., Minarovičová L., Kimličková V. (2016). Effects of white grape preparation on sensory quality of cookies. Acta Chim. Slovaca.

[11] The Association of Official Analytical Chemists (2000). AOAC (2000) Office Methods of Analysis.

[12] Ajila C., Leelavathi K., Rao U.P. (2008). Improvement of dietary fiber content and antioxidant properties in soft dough biscuits with the incorporation of mango peel powder. J. Cereal Sci..

[13] Singleton V.L., Rossi J.A. (1965). Colorimetry of total phenolics with phosphomolybdic-phosphotungstic acid reagents. Am. J. Enol. Vitic..

[14] Vamanu E., Nita S. (2013). Antioxidant capacity and the correlation with major phenolic compounds, anthocyanin, and tocopherol content in various extracts from the wild edible Boletus edulis mushroom. BioMed Res. Int..

[15] Papoutsis K., Pristijono P., Golding J.B., Stathopoulos C.E., Bowyer M.C., Scarlett C.J., Vuong Q.V. (2018). Optimizing a sustainable ultrasound-assisted extraction method for the recovery of polyphenols from lemon by-products: Comparison with hot water and organic solvent extractions. Eur. Food Res. Technol..

[16] Apak R., Güçlü K., Özyürek M., Karademir S.E. (2004). Novel total antioxidant capacity index for dietary polyphenols and vitamins C and E, using their cupric ion reducing capability in the presence of neocuproine: CUPRAC method. J. Agric. Food Chem..

[17] Kohajdová Z., Karovičová J., Magala M., Kuchtová V. (2014). Effect of apple pomace powder addition on farinographic properties of wheat dough and biscuits quality. Chem. Pap..

[18] Sudha M.L., Baskaran V., Leelavathi K. (2007). Apple pomace as a source of dietary fiber and polyphenols and its effect on the rheological characteristics and cake making. Food Chem..

[19] Alongi M., Melchior S., Anese M. (2019). Reducing the glycemic index of short dough biscuits by using apple pomace as a functional ingredient. LWT.

[20] Ratcliff R.K. (1977). Nutritional Value of Sunflower Meal for Ruminants.

[21] Hidalgo A., Brandolini A., Čanadanović-Brunet J., Ćetković G., Šaponjac V.T. (2018). Microencapsulates and extracts from red beetroot pomace modify antioxidant capacity, heat damage and colour of pseudocereals-enriched einkorn water biscuits. Food Chem..

[22] Lomascolo A., Uzan-Boukhris E., Sigoillot J.C., Fine F. (2012). Rapeseed and sunflower meal: A review on biotechnology status and challenges. Appl. Microbiol. Biotechnol..

[23] Vaher M., Matso K., Levandi T., Helmja K., Kaljurand M. (2010). Phenolic compounds and the antioxidant activity of the bran, flour and whole grain of different wheat varieties. Procedia Chem..

[24] Srivastava S., Genitha T., Yadav V. (2012). Preparation and quality evaluation of flour and biscuit from sweet potato. J. Food Process Technol..

[25] Bhat M., Hafiza A. (2016). Physico-chemical characteristics of cookies prepared with tomato pomace powder. J. Food Process. Technol..

[26] Chauhan A., Saxena D., Singh S. (2016). Physical, textural, and sensory characteristics of wheat and amaranth flour blend cookies. Cogent Food Agric..

[27] Gandhi A., Kotwaliwale N., Kawalkar J., Srivastav D., Parihar V., Nadh P.R. (2001). Effect of incorporation of defatted soyflour on the quality of sweet biscuits. J. Food Sci. Technol..

[28] Gallagher E., Kenny S., Arendt E.K. (2005). Impact of dairy protein powders on biscuit quality. Eur. Food Res. Technol..

[29] De Toledo N.M.V., Nunes L.P., da Silva P.P.M., Spoto M.H.F., Canniatti-Brazaca S.G. (2017). Influence of pineapple, apple and melon by-products on cookies: Physicochemical and sensory aspects. Int. J. food Sci. Technol..

[30] EFSA EU Register on Nutrition and Health Claims. https://ec.europa.eu/food/safety/labelling_nutrition/claims/nutrition_claims_en.

[31] Piluzza G., Bullitta S. (2011). Correlations between phenolic content and antioxidant properties in twenty-four plant species of traditional ethnoveterinary use in the Mediterranean area. Pharm. Biol..

[32] Gbenga-Fabusiwa F.J., Oladele E.P., Oboh G., Adefegha S.A., Oshodi A.A. (2018). Polyphenol contents and antioxidants activities of biscuits produced from ginger-enriched pigeon pea–wheat composite flour blends. J. Food Biochem..

[33] Aksoylu Z., Çağindi Ö., Köse E. (2015). Effects of blueberry, grape seed powder and poppy seed incorporation on physicochemical and sensory properties of biscuit. J. Food Qual..

[34] Martins S., Jongen W., van Boekel M. (2001). A review of Maillard reaction in food and implications to kinetic modelling. Trends Food Sci. Technol..

[35] Naknaen P., Itthisoponkul T., Sondee A., Angsombat N. (2016). Utilization of watermelon rind waste as a potential source of dietary fiber to improve health promoting properties and reduce glycemic index for cookie making. Food Sci. Biotechnol..

[36] Davidov-Pardo G., Moreno M., Arozarena I., Marín-Arroyo M., Bleibaum R., Bruhn C. (2012). Sensory and consumer perception of the addition of grape seed extracts in cookies. J. Food Sci..

